# Comparative Evaluation of the Powder and Tableting Properties of Regular and Direct Compression Hypromellose from Different Vendors

**DOI:** 10.3390/pharmaceutics15082154

**Published:** 2023-08-17

**Authors:** Nihad Mawla, Maen Alshafiee, John Gamble, Mike Tobyn, Lande Liu, Karl Walton, Barbara R. Conway, Peter Timmins, Kofi Asare-Addo

**Affiliations:** 1 Department of Pharmacy, University of Huddersfield, Huddersfield HD1 3DH, UK; nihad.mawla@hud.ac.uk (N.M.); maen.pharm@gmail.com (M.A.); b.r.conway@hud.ac.uk (B.R.C.); 2 Drug Product Development, Bristol Myers Squibb, Moreton, Merseyside CH46 1QW, UK; john.gamble@bms.com (J.G.); mike.tobyn@bms.com (M.T.); 3 Department of Chemical Sciences, University of Huddersfield, Huddersfield HD1 3DH, UK; l.liu@hud.ac.uk; 4 EPSRC Future Metrology Hub, University of Huddersfield, Huddersfield HD1 3DH, UK; k.walton@hud.ac.uk

**Keywords:** hypromellose, direct compression, controlled release, alternate sourcing, particle characteristics, density, compaction, compressibility, tabletability

## Abstract

Hypromellose, a widely used polymer in the pharmaceutical industry, is available in several grades, depending on the percentage of substitution of the methoxyl and hydroxypropyl groups and molecular weight, and in various functional forms (e.g., suitable for direct compression tableting). These differences can affect their physicomechanical properties, and so this study aims to characterise the particle size and mechanical properties of HPMC K100M polymer grades from four different vendors. Eight polymers (CR and DC grades) were analysed using scanning electron microscopy (SEM) and light microscopy automated image analysis particle characterisation to examine the powder’s particle morphology and particle size distribution. Bulk density, tapped density, and true density of the materials were also analysed. Flow was determined using a shear cell tester. Flat-faced polymer compacts were made at five different compression forces and the mechanical properties of the compacts were evaluated to give an indication of the powder’s capacity to form a tablet with desirable strength under specific pressures. The results indicated that the CR grades of the polymers displayed a smaller particle size and better mechanical properties compared to the DC grade HPMC K100M polymers. The DC grades, however, had better flow properties than their CR counterparts. The results also suggested some similarities and differences between some of the polymers from the different vendors despite the similarity in substitution level, reminding the user that care and consideration should be given when substitution is required.

## 1. Introduction

Hypromellose (hydroxypropyl methylcellulose, HPMC) is a non-ionic, semi-synthetic, inert, viscoelastic hydrophilic polymer existing as a creamy white powder that is tasteless and odourless [1]. It is composed of o-methylated and o-(2-hydroxyl propylated) cellulose, produced from treating alkali cellulose with propylene oxide and chloromethane. Various grades of HPMC are available with differences in their methoxyl and hydroxypropoxyl substitution, viscosity, molecular weight, and particle size distribution. Differences in the percentages of methoxyl and hydroxypropoxyl groups provide for the grade name (e.g., “K” or “E”) or equivalent substitution type (2208 or 2910, respectively) describing the polymer [2]. HPMC is the most widely used cellulose ether in the pharmaceutical industry and has been used as a tablet disintegrant, binder, viscosity-increasing agent, and in extended-release (ER) technology [3,4,5]. It is also used as a suspending and thickening agent in liquid pharmaceuticals [6].

Particle size is one of the essential factors of a material or formulation that can affect the mechanical strength of tablets produced from them. A decrease in the particle size of the powder may result in an increase in tablet tensile strength, especially for plastically deforming materials. Generally, a small particle size allows for a high surface area for bonding, resulting in increased tensile strength under equivalent compaction conditions relative to larger particles. Large particles, which deform via plastic deformation, tend to make weaker tablets compared to small particles, but for brittle materials, this may be less pronounced due to particle fracture under applied pressure creating a new surface for bonding [7].

It is also suggested that compressibility profile, tensile strength, and elastic recovery are affected significantly by the particle size of the HPMC polymer, as it exhibits plastic deformation under compaction [8]. Additionally, powder surface area and particle shape were more important than the degree of the substitution in respect of the compaction behaviour of the polymers [9]. The effect of viscosity grades on the profile of drug release and compaction behaviour has also been investigated. It was observed that the highest viscosity grade polymer studied (HPMC K100M) formed tablets with higher tensile strength compared to the other viscosity grades of the same polymer [10]. It was confirmed that at compression speeds between 15 to 500 mm/s and different compression forces between (5 to 20 kN), HPMC K100M was the more compressible polymer of those studied due to its plasticity in comparison to other grades evaluated [10]. 

Investigations into the compaction and flow properties of different HPMC grades have confirmed that all non-direct compression grades of HPMC polymers display poor to very poor flowability compared to their direct compression (DC) grades, which showed better flowability due to it having a more rounded, smoother surface and larger particle size [6]. The DC grades of HPMC are made in different processes. According to the Dow Chemical Company, Methocel DC2 polymer is prepared by a process in which the polymer particles are physically modified to reduce fines and enhance the spherical nature of the particles, helping to improve the direct compression process by optimising the powder’s flowability whilst maintaining the other important properties of their controlled-release Methocel regular (CR) grade counterpart [6]. The Ashland Chemical Company manufactures its Benecel DC grade via a co-processing process. Here, the HPMC is co-processed to coat it with silicon dioxide to improve the flowability of the polymer [11,12,13]: the properties of different viscosity grades of this specific DC material have been described relative to the regular material from this manufacturer [12]. Co-processing of MCC with silicon dioxide has been shown to improve the flow and also the compressibility of that material, so it may be logical to consider that it could do so for HPMC [14,15]. 

The high molecular weight K (HPMC 2208, USP) and E (HPMC 2910, USP) chemistries are frequently used polymers in ER matrix formulations [16] and hence this research focuses on the K chemistry grade of HPMC, specifically the 100,000 mPa.s (K100M) viscosity grades, because they may be preferred in extended-release hydrophilic matrix tablets [17]. Here, the K100M chemistry grades from four different vendors are investigated. Furthermore, the DC counterpart grades, where available, are also investigated to determine if their properties associated with tablet manufacture are equivalent to those for the related CR grades.

It is imperative to ensure that the sources of raw excipients used in manufacturing provide materials of essentially the same characteristics to ensure that the manufacturing process is sufficiently robust to incorporate materials from different sources. This is a key part of quality by design (QbD) and is also necessary for a robust business process. Having only a single source of a material makes a process vulnerable to supply chain shocks, such as the sudden absence from the market of a preferred vendor source. 

It is with this motive that the research described in this paper aims to extend prior work to determine if the K100M HPMC grades from different suppliers can be considered to be interchangeable with respect to the drug product manufacturing process. Given that chemistry and viscosity grades are equivalent, it could be considered that the polymer hydration and consequent drug release rate will be equivalent, hence, that behaviour has not been investigated in this work. Recently, the mechanical properties of HPMC from K4M and K100M from three vendors have been investigated, with a focus on compression speed [18]. The DC and CR grades were investigated for one vendor only (Methocel grades of K4M and K100M). The authors found the Benecel and Methocel K100M grades had the best compaction properties [18]. The material attributes of HPMC from two different vendors with a focus on viscosity grades (K4M, K15M, and K100M) have also been explored. Using principal component analysis (PCA) and orthogonal partial least squares discriminant analysis (OPLS-DA), the powder and tableting properties were recognised as the differential material attributes for the HPMC samples studied [19]. 

In this present study, we have investigated similarities and differences between the K100M HPMC grades from four different vendors as well as the different grades of the same polymer from the same supplier (such as DC, CR, and XR) by probing properties such as their flow, compactability, mechanical strength, and an in-depth analysis of their particle properties. This is to ultimately inform a formulator with regard to potential interchangeability between the various grades.

## 2. Materials and Methods

### 2.1. Materials

HPMC 2208 100,000 mPa.s materials were kindly provided by Colorcon Ltd., Kent, UK (Methocel^TM^ K100M CR and DC2, referred to as DC from henceforth); Ashland Industries Europe GmbH, Schaffhausen, Switzerland (Benecel^TM^ K100M PH DC, Benecel^TM^ K100M PHARM CR and Benecel^TM^ K100M PHARM XR); Biogrund GmbH, Neukirchner, Hünstetten, Germany (BonuCel^®^ D 100000 H 2208); and Shin-Etsu Chemical Co., Ltd., Tokyo, Japan (Metolose^®^ 90SH-100000 and Metolose^®^ 90SH-100000SR). Materials obtained were typical commercial materials, not specially selected lots, and so were considered representative materials from each vendor for comparative purposes. Exploring lot-to-lot variability from each vendor was not within the scope of the current study.

### 2.2. Micrometric Properties of the Polymers

#### 2.2.1. Scanning Electron Microscopy (SEM) and SEM with EDX

The morphology of the polymers was investigated using a scanning electron microscope (SEM) (Quanta FEG 250, FEI, Hillsboro, OR, USA). A small amount of each sample was placed on a metal stub using the conductive double-sided adhesive tape and coated with gold using Quorum SC7620 Sputter Coater under vacuum. The electron acceleration voltage was 20 kV. The micrographs were taken under several magnifications (X30, 100, 500, and 1000) and analysed.

EDX analysis was performed using the Oxford Instruments Aztec software version 4.2 (Oxford Instrumemts, Oxford, UK) using the 80 mm X-Max detector to give the elemental composition of the samples. The powder samples were prepared initially by mounting them onto a small aluminium stub and a carbon sticker was used to hold the powder down, after which it was air dusted to remove any loose particles. The samples were then carbon coated using the Quorum Q150T carbon coater, which deposited approximately 20 nm of carbon onto the samples. The Hitachi SU8230 was used for imaging a consistent range of magnifications with the settings optimised for each sample. 

#### 2.2.2. Particle Characterisation

Particle size characterisation was conducted by static image analysis using a fully automated image analysis light microscope particle characterisation system (Morphologi G3, Malvern Panalytical, Malvern, UK). The samples were dry dispersed onto a glass plate using the integrated Morphologi G3 solids dispersion unit, employing a dispersion pressure of 1 bar, an injection time of 20 ms and a settling time of 180 s. All samples were imaged using a 5× magnification lens (6.5–420 µm resolution range), taking 2 addition planes of focus above the plane of focus (equivalent to 97.8 µm) to account for 3-dimensionality within the sample. Due to the fibrous nature of the particles, the analysis was conducted using the fibre option. Morphological filters and settings for all samples were optimised on a sample-by-sample basis to account for differences in particle morphology. An addition to this was a pixel number filter to remove all particles consisting of fewer than 100 pixels, which was applied for the purposes of shape analysis.

#### 2.2.3. Bulk Density, Tapped Density, and True Density of the Polymers

The bulk volume (V_0_) of each polymer sample was calculated by gently weighing the powder sample (W_s_) into a 10 mL dry graduated cylinder. The cylinder was then manually tapped 150 times to calculate the tapped volume (V_f_) of the powder. All the measurements were conducted in triplicate. The bulk density (ρ*_bulk_*) and tapped density (ρ*_tapped_*) were calculated using Equations (1) and (2).

(1) $$\rho_{\mathrm{bulk}}=\frac{W_{s}}{V_{0}}$$

(2) $$\rho_{\mathrm{tapped}}=\frac{W_{s}}{V_{f}}$$

Carr’s index (CI) was calculated using Equation (3) [20].

(3) $$\mathrm{CI}\;(\%)=\frac{\rho_{bulk}-\rho_{tapped}}{\rho_{bulk}}\times 100$$

The true density of the HPMC polymers was obtained using a gas pycnometer with helium as the displacement gas (AccuPyc II 1340 Gas Pycnometer, Micromeritics, Hitchin, UK). Each polymer was accurately weighed (1.5–2 g) and placed gently in the sample cell. The result of the mean and standard deviation of three runs was obtained.

#### 2.2.4. Flowability Using a Ring Shear Tester

The flow properties of these polymers were also characterised using a ring shear method (RST-XS, Dietmar Schulze, Wolfenbuttel, Germany). A specific cell was used where the powder filled in. The weight of the filled cell was recorded and entered onto the software provided. Then, a pre-shear stress of (4000 Pa), followed by 25%, 38%, 51%, 65%, and 25% of the specified pre-shear stress was applied. The flow function coefficient (FFC) for each powder was then calculated using Equation (4) [21,22]:

(4) $$FFC=\frac{\sigma_{c}}{\sigma_{u}}$$

where $\sigma_{c}$ is the consolidation stress that compacts the beds and $\sigma_{u}$ represents the unconfined yield stress that makes the powder bed flow. FFC values can range from less than 1 to more than 10, with flowability increasing as FFC increases [22]. In addition to flow characterisation, wall friction interaction with stainless steel coupons was investigated using the RST.

### 2.3. Compact Preparation

Flat-face 10 mm cylindrical tablets were prepared using unlubricated stainless-steel die single-sided compaction with polished stainless-steel dies of circular cross-section (5.00 mm internal radius) and closely-fitting flat-faced punches (Specac Limited, Kent, UK). Compaction was applied at five different pre-set peak forces (F_max_) of (5, 7.5, 10, 12.5, and 15 kN) using a computer-controlled mechanical testing machine (M500-50CT, Testometric Company Ltd., Rochdale, UK), attached with compression platens. The lower punch was inserted into the die, followed by the addition of a pre-weighed amount of powder (approximately 250 mg). The upper punch was inserted with gentle pressure to achieve a visually uniformly packed powder bed as starting compaction point. The filling depth (*h*_0_) was recorded at the starting crosshead position of the powder bed. The compaction speed was set at 3 mm min^−1^ throughout the compression period to the chosen maximum loading. The force (*F*) was applied through the push-rod between the upper punch and the load cell. Whilst the lower punch remained stationary. The displacement of the upper punch was recorded using a linear variable differential transformer (LVDT) position gauge probe (Solartron AX/2.5/S, Solartron Metrology Limited, Portsmouth, UK) linked to the platens. As soon as the required peak force was achieved, the upper punch was allowed to retract at 1 mm min^−1^ [23,24]. The tablet was gently ejected from the die using the compression rig. The length (*h*_T_) and the diameter (d) of the tablet were determined using an electronic digital calliper (Mitutoyo, Japan) 1 min after tablet ejection from the die, and the mass (m) of the tablet was determined subsequently. 

### 2.4. Compactability Analysis

Analysing the deformation behaviour of each excipient in the prepared compact is critical. For “in-die” or “at pressure” approach, the dimensions of the tablets are calculated using apparatus like a compaction simulator or instrumented tablet press. While for “out-of-die” or “zero-pressure” approach, the tablet dimensions were calculated after ejection. The in-die approach is generally the most useable method due to the ease of data collection and generation of faster results [25]. 

For the “in-die” analysis, the force *F*(t) and displacement *x*(t), as a function of time, were measured at 0.2 s intervals during the test and were recorded automatically. (*h*_0_-displacement) was calculated using the highest displacement recoded by the apparatus plus the height of the tablet after ejection; this was used subsequently to correct the displacement that was recorded from the apparatus. The upper punch pressure was calculated using Equation (5),

(5) $$P(t)=\frac{F(t)}{\pi R^{2}}$$

and the relative density was calculated using Equation (6); m is the mass of the tablet after ejection,

(6) $${Ρ}_{\mathrm{rel}}(t)=\frac{m}{\rho \pi R^{2}[h_{0}-x\left(t\right)]}$$

“Out-of-die” analysis: After 24 h following tablet ejection, the weight, diameter, thickness, and hardness were measured for each tablet. The compaction pressure can be calculated using Equation (7),

(7) $$\mathrm{Compaction}\;\mathrm{pressure}=\frac{Force}{Area}$$

The tensile strength can be calculated using Equation (8), where H is the hardness, D is the diameter, and T is the thickness of the tablet,

(8) $$\mathrm{Tensile}\;\mathrm{strength}=\frac{2H}{\pi DT}$$

Solid fraction was calculated using Equation (9), where tablet density was calculated from the volume and weight of the tablet,

(9) $$\mathrm{Solid}\;\mathrm{fraction}=\frac{Tablet\;density}{True\;density}$$

Tablet porosity, the total volume occupied by the air inside the tablet, was calculated using Equation (10),

(10) $$\mathrm{Porosity}=1-\frac{Tablet\;density}{True\;density}$$

Axial expansion, which gives information on the lamination tendency of the tablet on storage, was calculated from the tablet thickness after 1 min (*h*_c_) and 24 h (*h*) after compression using Equation (11) [25,26],

(11) $$\mathrm{Axial}\;\mathrm{expansion}\;(\%)=(\frac{h-h_{c}}{h_{c}})\times 100$$

### 2.5. Factor Analysis

To have an overall perspective of the similarities between the materials measured, a principal component analysis (PCA) based approach called factor analysis was used [27]. This method differs from the PCA by having axes rotated such that a better judgement can be made. The main purpose of the factor analysis in this article is to identify the groups of the materials to which they belong in terms of their measured properties and compaction force. 

The measured properties are: 

1—compression stress, 2—solid fraction, 3—relative tensile strength (RTS), 4—applied density, 5—hardness, 6—tablet volume, 7—tablet thickness, 8—uncompacted density, 9—density at maximum pressure, 10—final density, 11—maximum density, and 12—elastic recovery. 

The materials are referred to as: 

M1—Benecel CR, M2—Benecel XR, M3—Benecel DC, M4—Methocel CR, M5—Methocel DC, M6—Metolose 90SH, M7—Metolose 90SH SR and M8—BonuCel D. 

Each of the materials underwent an applied compaction force of 15 kN, 12.5 kN, 10 kN, 7.5 kN, and 5 kN. The data structure for the factor analysis was then constructed. A three-dimensional data matrix was formed to cover the individual materials, the measured properties, and the applied compression forces where Mi was used to refer to material i, and j used to refer to property number j. The properties (α values) were measured for each material at each compaction force and represented as α_ij,5_–α_ij,15_ (α_ij,5_, therefore, means the data for material i and property j under compaction force 5 kN and so forth).

The α values were then used in the computation for the factor analysis. Two factors, F1 and F2, were extracted from the computation of eigen values, with the first factor covering 89.1% data variation and the second covering 10.9%. The corresponding scores (SF1 on F1 and SF2 on F2) for each material on all the properties under each compaction force were also computed.

## 3. Results and Discussion

### 3.1. Particle Size and Shape of the Polymers

**Figure 1:**
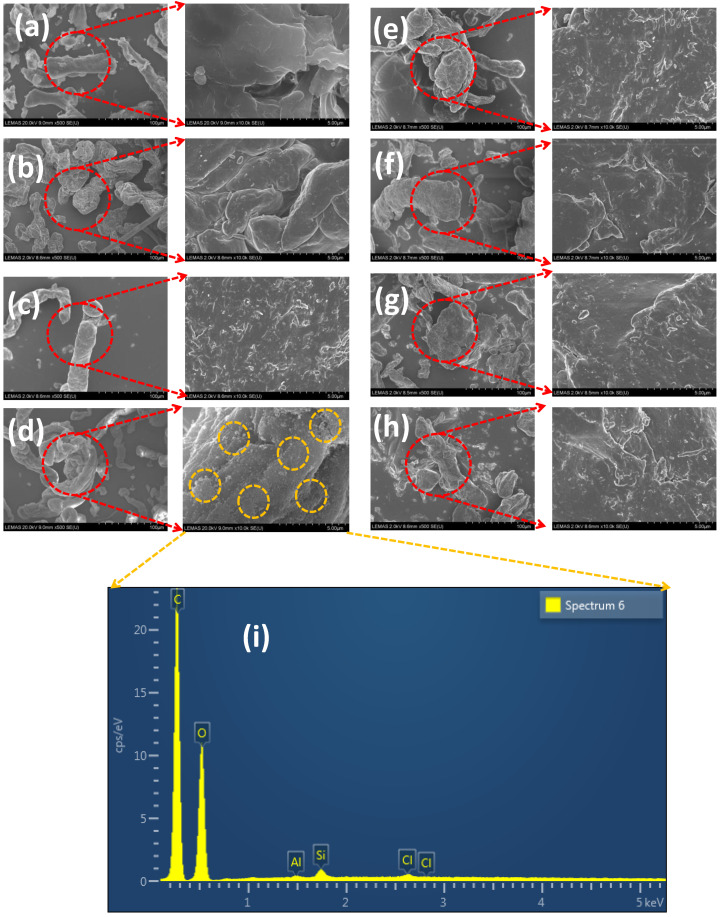
SEM photomicrographs of HPMC K100M from different vendors: (**a**) Methocel CR, (**b**) Methocel DC, (**c**) Benecel CR, (**d**) Benecel DC, (**e**) Benecel XR, (**f**) Metolose 90SH, (**g**) Metolose 90SH SR, and (**h**) Bonucel. EDX analysis image of (**i**) Benecel DC.

Methocel DC consisted of rounded filamentous particles, whereas the Methocel CR grade is composed of various sizes of flattened and angular particles with definite edges and a smaller particle size than the DC grade. The particles of the Methocel DC grade material appeared to be composed of agglomerated fibres similar to the individual particles seen in the CR grade material. It appears that the DC material may be manufactured by a process that agglomerates and densifies material like the CR grade to yield the DC grade particles. Indeed, Dow holds a patent for making granular HPMC employing an aqueous wet granulation process in a fluid bed granulator or a high-shear granulator [28]. It was apparent from the SEM images that the Methocel CR grade consisted of a larger proportion of fine particles compared to Methocel DC grade. Metolose 90SH, Metolose 90SH SR, Bonucel, Benecel DC, Benecel CR, and Benecel XR all had similar morphologies (Figure 1).

Using the Morphologi G3 instrumentation, the valuable size, shape, and agglomeration state data were acquired. Such data are of greater utility in describing particle characteristics and relating that to bulk behaviours than conventional particle size measurement [29]. All the HPMC samples tested were confirmed as fibre-like in nature with a tendency to form entwined bundles and/or some more tightly formed agglomerated assemblages (more so in the case of the Methocel samples). For the Methocel DC material, there was a larger population of median/large width particles (fewer fines) and a small (but reproducible) upward shift in the volume-weighted size distribution. It was, therefore, easy to distinguish any number of differences (size, fibre length, fibre width, and light transmission) between the Methocel CR and DC grades, and this could provide an explanation as to why the DC material could be expected to exhibit improved flow/handling (Supplementary Figure S1). The arithmetic mean size distribution for Methocel DC showed an apparent bimodal size distribution, whereas Methocel CR showed essentially a monomodal arithmetic size distribution with, interestingly, a wide distribution having a skewness to the coarser side of the distribution. The same is true for fibre length and fibre width. In comparison to the Methocel CR grade, the arithmetic size distribution for the Methocel DC grade showed a reduction in the population size of particles in the sub ten µm range and a corresponding increase in the particle population in the 10–100 µm range. This shift in the size of the fine particle fraction was also observed in the shape data, where the average length and width of the low-volume particles was observed to increase.

The equivalent volume-weighted distributions show no significant change in fibre length; however, the width of the fibres was observed to increase. For the DC grade, this bimodal distribution might suggest the presence of a population of a coarse fraction of particles, which enables the flow of the whole distribution by acting as a glidant, impairing particle–particle interaction within the fines fraction, and reducing their impact on cohesive behaviour of the bulk. Similar observations have been made in comparing the properties of fine and coarse particle size grades of microcrystalline cellulose [30]. In summary, the data shows that whilst the size is generally similar for the two grades, the size of the fines population were different. The small shift in the volume-weighted size can be seen to correspond to the increased particle width of the high-volume particles. This seems to correspond to the vendor’s claims of “reduced fines”, but the size and morphological nature of the particles also appear to be different in the DC material, which may contribute to improved flow properties.

For the Benecel CR/DC/XR samples, there was very little difference between the three samples (very slight differences in arithmetic size distribution, which was reproducible). The CR grade for Benecel was however equal/similar to its DC counterpart. All Benecel grades had a very similar apparent bimodal size distribution for the arithmetic size distribution, although the XR grade, however, appears to contain a larger relative population of particles in the 10–100 µm range corresponding to an increase in both length and width of particles in the low volume fraction. No such shift in shape was observed for the particles in the high volume fraction (Supplementary Figure S2). As a general rule, the Methocel samples were comparatively larger than the Benecel samples. The Metolose 90SH SR and Metolose 90SH samples were essentially identical to each other in geometric and arithmetic size, fibre length, and fibre width (Supplementary Figure S3), exhibiting an essentially monomodal arithmetic size distribution. The geometric and arithmetic size and fibre length and width for the Bonucel D HPMC is depicted in Supplementary Figure S4. Both the Metolose materials and the Bonucel material had a similar size distribution data to Methocel CR, although the arithmetic length and width distributions appeared to be narrower/less bimodal in nature

### 3.2. Flow Properties of the Polymers

**Figure 2:**
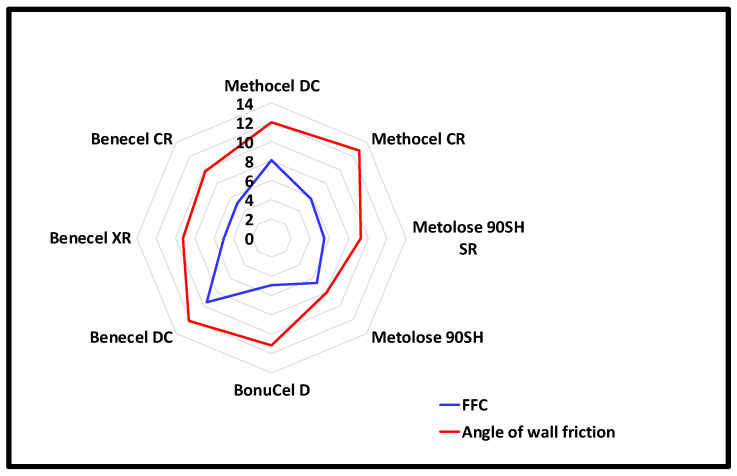
FFC values and angle of wall friction (°) values of different HPMC grades tested using stainless steel coupons on the ring-shear tester. Note: vertical black numbers represents the values for both FFC and angle of wall friction. Error bars are omitted for clarity; standard deviations were in the range of 1.2–27.7%.

**Figure 3:**
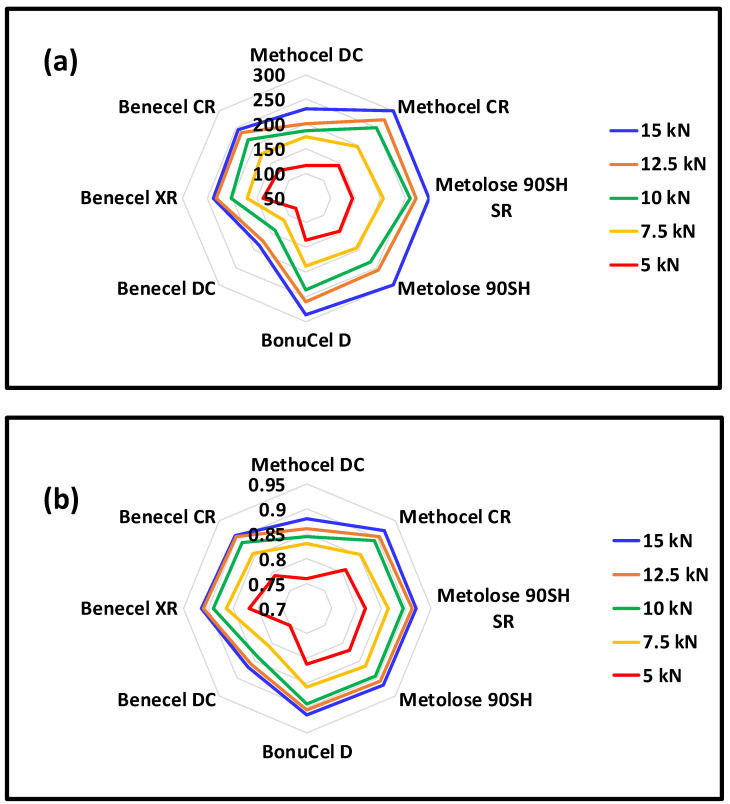
(**a**) Hardness (N) and (**b**) solid fraction data of the compacted polymer compacts from the various vendors after 24 h. Note: vertical black numbers represents the values for (**a**) hardness and (**b**) solid fraction. Error bars are omitted for clarity; standard deviations were in the range of 0.1–12.4% for hardness and 0.1–1.5% for the solid fraction.

The bulk (0.24–0.31 g/cm^3^), tap (0.36–0.45 g/cm^3^), and true densities (0.30–0.33 g/cm^3^) of all the tested polymers were relatively similar. These values were similar to that reported by others [18]. Bonucel had the lowest bulk density (0.24 g/cm^3^), followed closely by the Methocel DC and CR grades (0.25 g/cm^3^). Benecel DC had the highest bulk density (0.31 g/cm^3^). The lowest tapped density was experienced by the Methocel DC grade polymer (0.36 g/cm^3^), whereas Benecel DC and Metolose 90SH experienced the highest tap density (0.45 g/cm^3^). Methocel DC and Benecel DC grades had Carr’s index values of 30% for both, demonstrating better flowability than the other tested polymers, which had Carr’s index values ranging from 38–40. Using the ring-shear testing instrument, the HPMC grades showed a relatively similar bulk density, ranging between 0.326 and 0.385 g/cm^3^) (Figure 2). However, FFC values of these grades were significantly different, with DC grades having higher FFC values in comparison to the other grades. Benecel DC had an FFC value of 9.5, while Methocel DC had an FFC value of 8.05, indicating that Benecel DC has comparatively better flowability than Methocel DC. Methocel CR, Benecel CR, Benecel XR, Metolose 90SH SR, and BonuCel D all had similar FFC values of ~6, indicating that these grades have similar flowability profiles (Figure 3). Metolose 90SH, however, displayed a relatively higher FFC value of 6.65. The angles of wall friction of these grades are presented in Figure 2. The values for the angles of wall friction (9°–13°) obtained suggest that these grades will behave similarly in any interaction with a stainless steel surface.

### 3.3. Hardness and Solid Fraction of Polymer Compacts

Figure 3a,b depicts the respective hardness and solid fraction profiles of tablets that have been compressed at forces ranging between 5 to 15 kN. Hardness values were highly dependent on the applied compaction force used to produce the tablet, the higher the compaction force and the harder the tablet. At the lowest compression force of 5 kN, Metolose 90SH, Metolose 90SH SR, Methocel CR, Benecel XR, BonuCel D, and Benecel CR produced relatively stronger tablets ranging from 129 to 145 N with solid fractions ranging from 0.79 to 0.82. (Figure 3a,b). This may be related to the particle size and shape of the polymer and the manufacturing process

The Methocel DC and Benecel DC polymers had relatively lower hardness values (115.3 and 78.2, respectively) with solid fractions of 0.75 and 0.7. At the higher compression force of 15 kN, Methocel CR, Metolose 90SH SR, Metolose 90SH, BonuCel D, Benecel CR, Benecel XR, and Methocel DC produced compacts with high hardness values (231–300 N) with a higher solid fraction (0.88–0.91) (Figure 3a,b). Benecel DC, however, produced relatively weaker compacts (186 N) with the lowest solid fraction value (0.86) in comparison to all other polymers. A comparison of the CR and DC grades of Methocel and Benecel polymers shows that the CR grades outperform the DC in that they provide for harder compacts and a greater solid fraction for a given applied force.

### 3.4. Tabletability and Compressibility of Polymers

**Figure 4:**
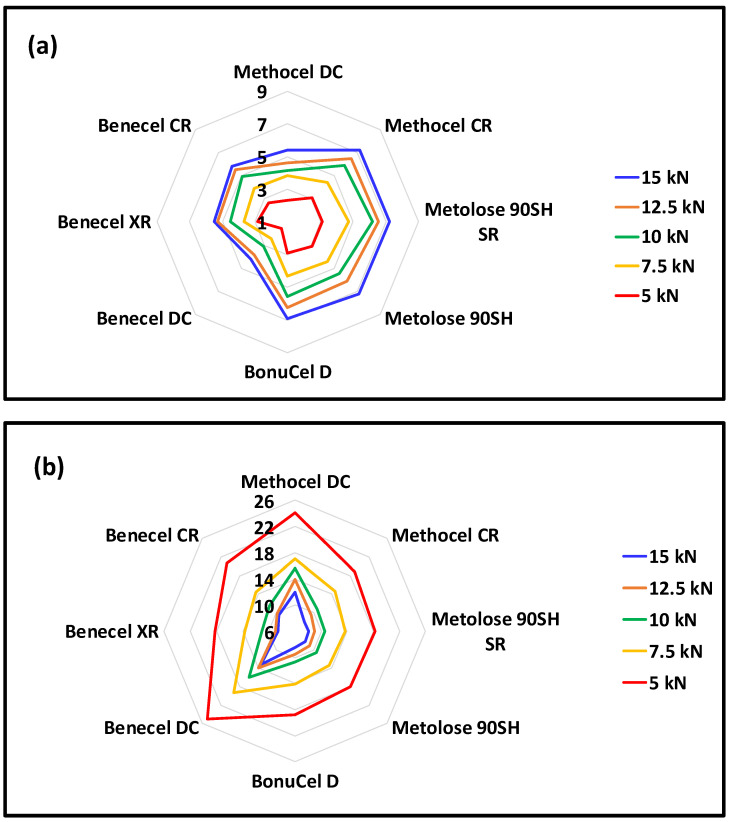
(**a**) Tabletability profile and (**b**) compressibility profile (kN) of HPMC powders from different vendors under five compression forces (5, 7.5, 10, 12.5, and 15 kN). Note: vertical black numbers represents the values for (**a**) tabletability and (**b**) compressibility. Error bars are omitted for clarity; standard deviations were in the range of 0.3–12.5% for tabletability and 0.1–1.5% for compressibility.

The relationship between compression stress and tensile strength is termed tabletability. It describes the performance of the applied pressure in increasing the tensile strength and the relationship between the compaction pressure and the strength of the tablet [31,32,33]. Figure 4a shows the tabletability profiles of the compacts (5 to 15 kN). The relative tensile strength of all compacts increased with increasing compaction stress. That is because a higher compaction stress led to a longer-lasting plastic deformation of the material particles, thereby sustaining a more significant bonding area between the neighbouring particles [34]. At the same compression stress, Methocel CR grade showed better tabletability than the Methocel DC grade. This was also true for the Benecel grades. Benecel CR and a Benecel XR grades showed better tabletability than Benecel DC grade at all the compression pressures studied. Metolose 90SH SR, however, displayed relatively similar tabletability as the Metolose 90SH grades. Broadly speaking, apart from the Methocel DC and Benecel DC grades, all the other polymers had similar tabletability profiles. The better performance of the CR grade of Methocel relative to the DC grade might relate to the greater population of finer particle fraction in the CR grade of this plastically deforming material, whereby a greater surface area for particle–particle bonding will be available. The poorer tableting performance of the DC grade of Benecel is not related to the relative size of the population of finer particles, as the grades are more or less comparable in this parameter. The less effective tableting may be associated with the co-processing of the polymer with silicon dioxide technology used to prepare Benecel DC, with the silicon dioxide potentially covering some of the sites available for particle–particle bonding and impairing this aspect of compact formation.

**Figure 5:**
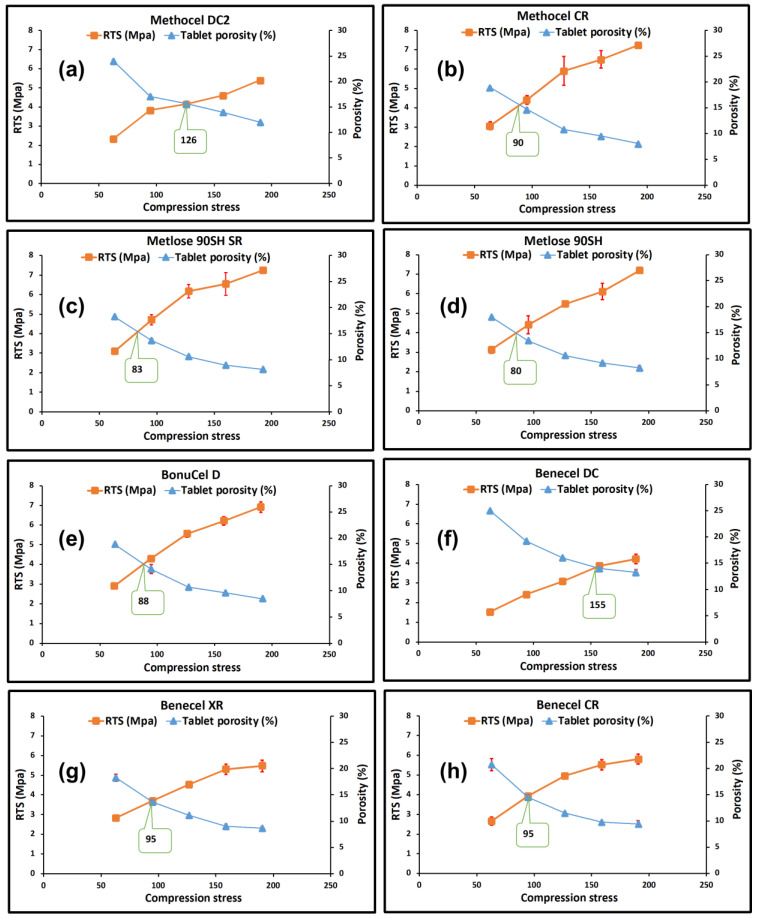
Compaction properties of HPMC powders from different vendors. Relative tensile strength (RTS) (MPa) and porosity (%) relationship with compression stress (MPa) representing the respective tabletability and compressibility profiles at the same compression force (error bars are ±SD) for (**a**) Methocel DC, (**b**) Methocel CR, (**c**) Metlose 90SH SR, (**d**) Metlose 90SH, (**e**) BonuCel D, (**f**) Benecel DC, (**g**) Benecel XR, and (**h**) Benecel CR.

**Figure 6:**
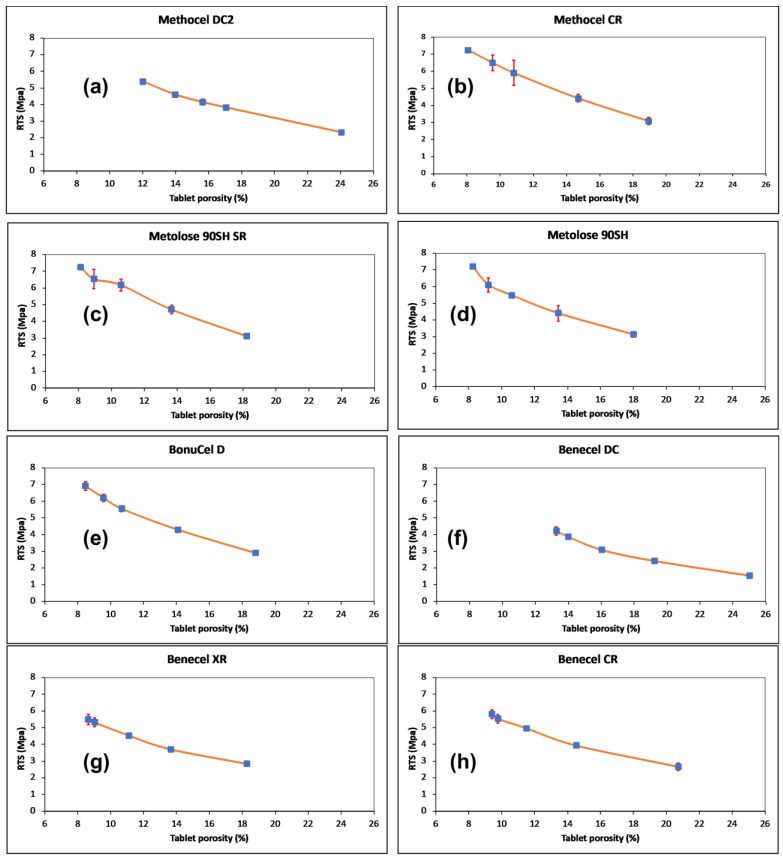
Compactibility of HPMC powders. Relative tensile strength (MPa) vs. tablet porosity (%) at five different compression stresses (error bars are ±SD) (for (**a**) Methocel DC, (**b**) Methocel CR, (**c**) Metlose 90SH SR, (**d**) Metlose 90SH, (**e**) BonuCel D, (**f**) Benecel DC, (**g**) Benecel XR, and (**h**) Benecel CR.

Compressibility is the ability of a material to undergo volume reduction as the result of applied pressure. This is caused by powder particle rearrangement and plastic deformation when the applied pressure increases and the compact tablet porosity is decreased [31,32,34]. The results obtained for all HPMC K100M grade polymers at the same compression force is depicted in Figure 4b. It was observed that the tablet porosity gradually decreased with an increase in compression stress (Figure 5). The Methocel DC grade had a higher porosity than the Methocel CR grade at each point of compression stress. This indicates that the Methocel DC grade has low compressibility, potentially due to its relatively large particle size [33]. The porosity of Benecel DC was also higher than both the Benecel CR and Benecel XR grades, which indicated lower compressibility. This may be attributed to the addition of silica in the coprocessing process [11]. The co-processing method for making Ashland DC material is covered in a patent assigned to the company and describes combining hypromellose powder with silicon dioxide employing high-shear mixing and milling processing. The yellow dashed rings around Figure 1d indicate where the silica is evident from the EDX analysis imaging (Figure 1i) all across the polymer. This mixing process is distinct from the process used to make silicified microcrystalline cellulose, where the cellulose and the silicon dioxide are brought together by spray drying from an aqueous suspension [35]. The spray drying process combines these two components differently from a blending process, potentially altering the ability of particles to bind under pressure favourably. The lower compressibility profile of Benecel DC could also be from the higher axial expansion of the compact after the decompression. It was interesting to note that the tablet porosity was similar at all the points of compression stress between Metolose 90SH SR and Metolose 90SH, indicating that the packing of these powders was identical—a consequence of their similar shape and particle size, as previously discussed. The BonuCel D polymer behaved quite similarly to the CR grades of the tested compacts. Another observation was made between the tabletability and the compressibility data. When plotting the data together, there was an intersection at around 15% porosity for all polymer grades (Figure 5). An early intersection point (i.e., lower value) indicates that the polymer has better tabletability and compressibility properties than when the intersection points occur much later. Figure 6, therefore, again implies that Metolose 90SH SR, Metolose 90SH, BonuCel D, Methocel CR, Benecel XR, and Benecel CR (intersection points: 83–95) had better tabletability and compressibility when compared to Methocel DC and Benecel DC (intersection points: 126 and 155, respectively).

### 3.5. Out-of-Die and In-Die Compactability Analysis

**Figure 7:**
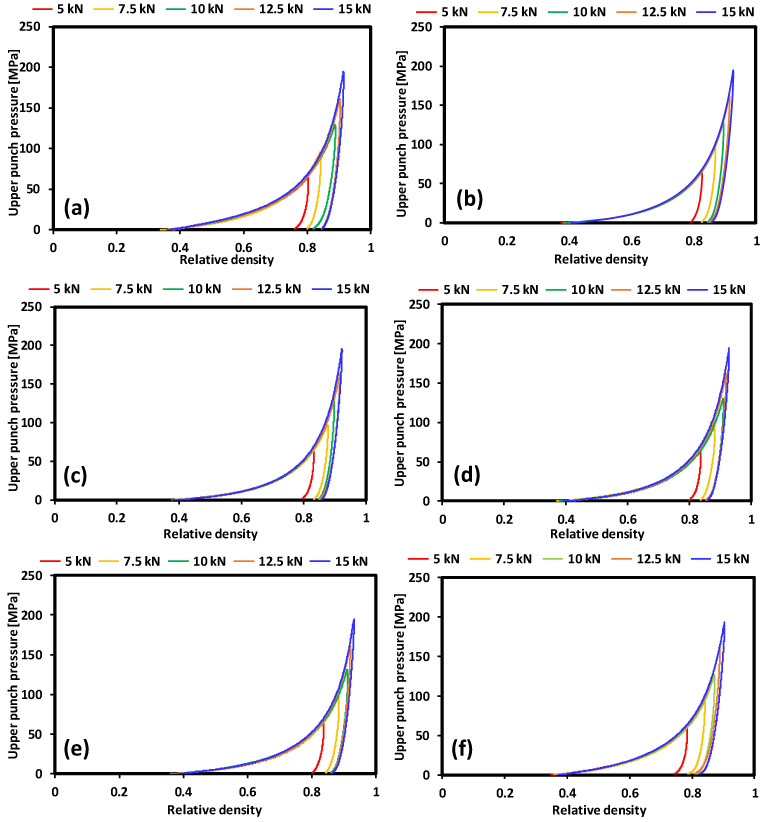

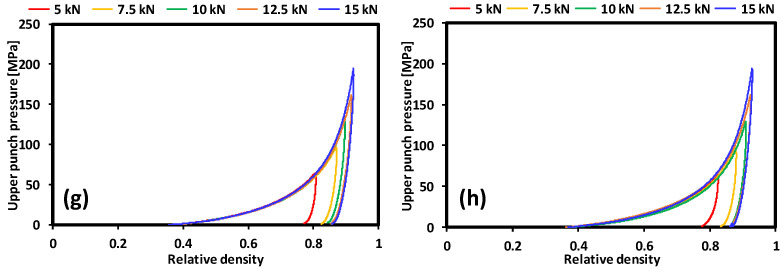
HPMC compaction curves of the polymers from the different vendors showing the relative density at various compaction pressures. (**a**) Methocel DC, (**b**) Methocel CR, (**c**) Metlose 90SH SR, (**d**) Metlose 90SH, (**e**) BonuCel D, (**f**) Benecel DC, (**g**) Benecel XR, and (**h**) Benecel CR.

Compactability is the ability of a powder to transform into a compact with sufficient strength during the process of densification. Compactability is presented in Figure 6 as the relationship between the tensile strength and the tablet porosity. The relative bonding strengths of the various polymers can be compared using a compactability plot, where at the same porosity, a higher tensile strength will indicate stronger bonding between the particles. A low porosity compact indicates higher tensile strength and a larger bonding area [31,32,33]. A general trend was observed for all the polymers from the various vendors. Tensile strength decreased with an increase in the porosities of the compacts. The Methocel DC grade exhibited higher porosity and, thus, lower tensile strength than its Methocel CR counterpart. This was also true for the Benecel grades. The Benecel DC grade displayed lower tensile strength and higher porosity compared to the Benecel XR and Benecel CR grades. These results, thus, indicate that the CR grades of both Methocel and Benecel had better compactibility profiles than their DC counterparts (Figure 6). The in-die compactibility behaviour of the HPMC K100M grades was determined by plotting the upper punch pressure versus the relative density of the powder during the loading and unloading processes (Figure 7). The upper punch pressure increased gradually and then rapidly through the loading stage. During the unloading stage, the relative density slightly decreased, indicating elastic recovery of the material, a general trend exhibited by all samples [23,24,36]. Elastic recovery occurred for the compacts, and this can lead to capping, delamination, and cracking of the compacted material. On the other hand, it may lead to excessive tooling wear because of the high friction forces produced during recovery [24,37,38]. 

### 3.6. Out-of-Die and In-Die Axial Expansion

**Figure 8:**
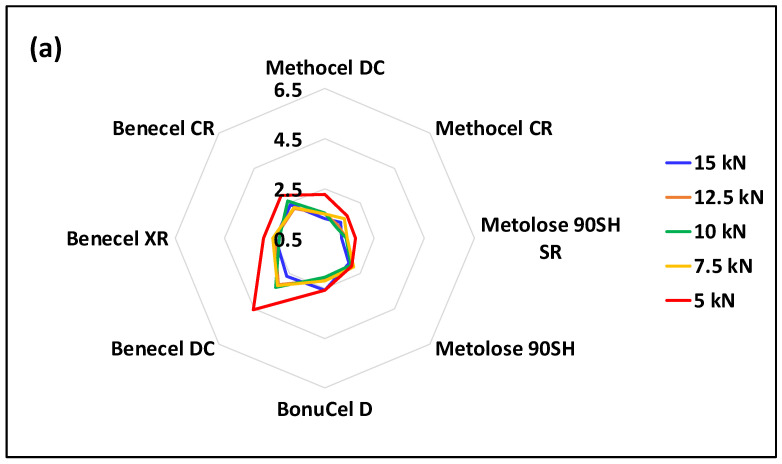

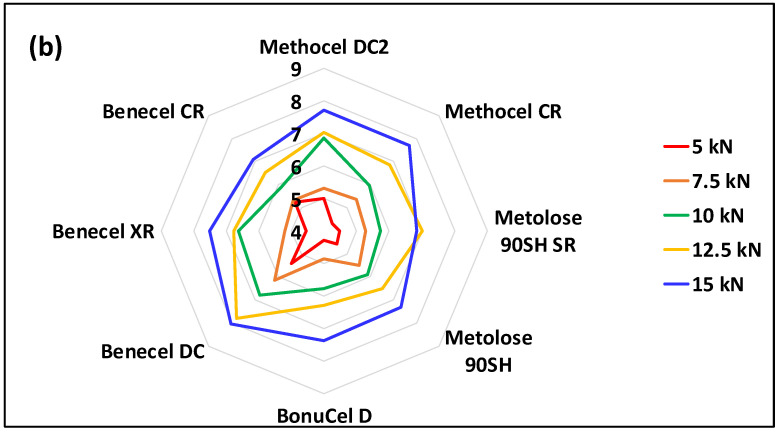
(**a**) In-die recovery (%) and (**b**) out-of-die axial expansion profile (%) of HPMC powders from different vendors under five compression forces. Note: vertical black numbers represents the values for (**a**) in-die recovery and (**b**) out-of-die axial expansion. Error bars are omitted for clarity; standard deviations were in the range of 4.2–7.6% for in-die recovery and 0.9–27.6% for out-of-die recovery.

Figure 8 shows the out-of-die axial expansion and in-die recovery for all the HPMC polymers tested. It can be observed in Figure 8a that the axial expansion did not increase significantly with increasing compaction pressure. For most of the polymers, the total axial expansion after 24 h was less than 3% at any applied pressure (Figure 8a). This low axial expansion may be related to the internal particle bonds and the plastic nature of the polymer [25]. The total axial expansion, however, for the Benecel DC grade after 24 h was relatively higher than the rest of the polymers tested and can be attributed to the formulation process used for the Benecel DC polymer compared to the Benecel XR and Benecel CR polymers. The use of silica in the manufacturing of Benecel DC [11] may have impaired the interparticle bonding ability of the polymer. Figure 8b depicts the in-die recovery, and although expansion is minimal at low compaction pressures, there is an increase with an increase in compaction pressure. It was interesting to note that the Benecel DC and Methocel DC polymers displayed relatively higher in-die recovery values compared to the similar behaviour of all other polymers (Figure 8b).

### 3.7. Factor Analysis

**Figure 9:**
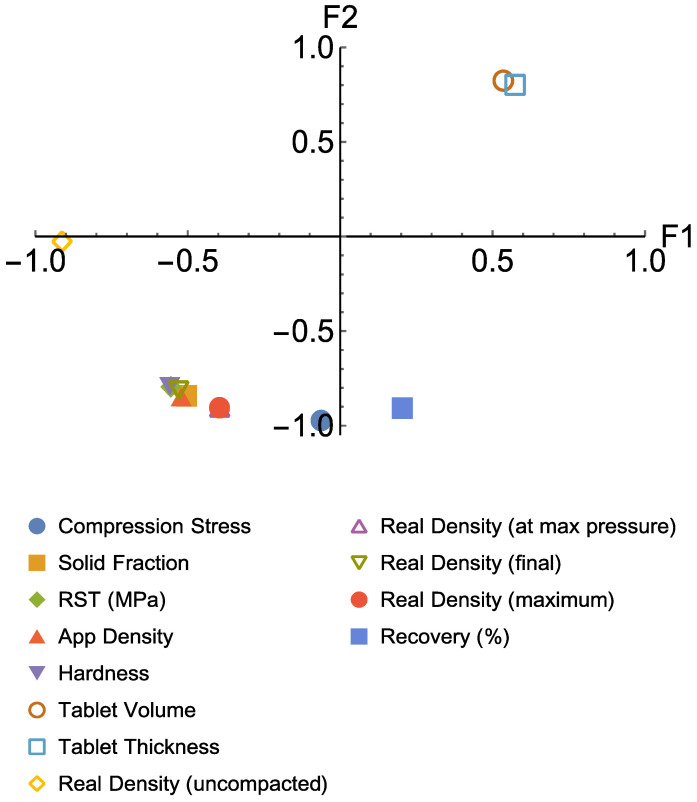
A plot of F2 vs. F1 showing the similarity between the measured properties.

**Figure 10:**
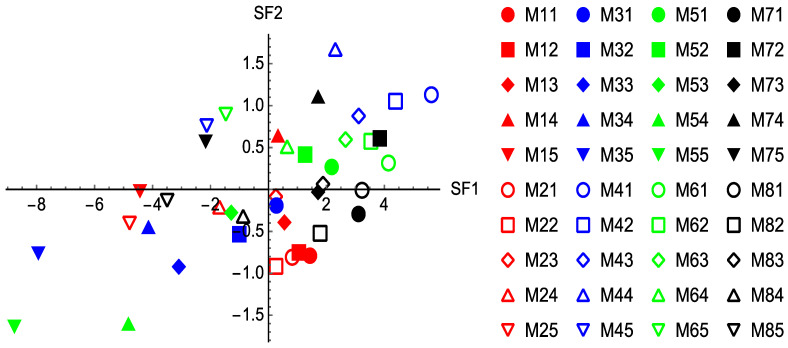
A plot of the scores of all the material-associated properties for each compaction force on F2 vs. that on F1. Note, in the legend, the first number on the right of M indicates the material: 1—Benecel CR, 2—Benecel XR, 3—Benecel DC, 4—Methocel CR, 5—Methocel DC, 6—Metolose 90SH, 7—Metolose 90SH SR, and 8—BonuCel D. The second number on the right of M indicates the compaction force: 1—15 kN, 2—12.5 kN, 3—10 kN, 4—7.5 kN, and 5—5 kN.

**Figure 11:**
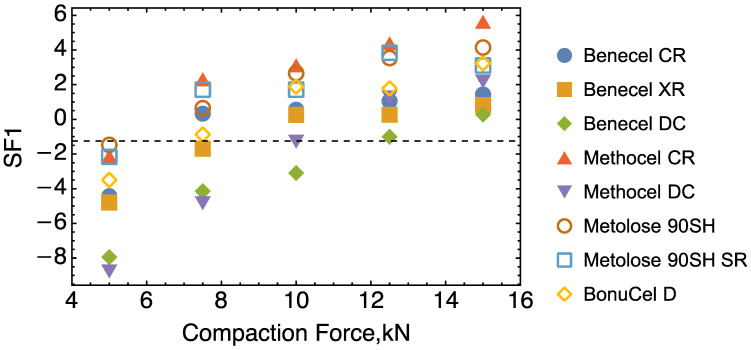
A plot of scores on F1 showing how the material properties change with the compaction force.

The plot of F2 vs. F1 (Figure 9) indicates the similarity between the material properties if they fall into the same quadrant. As can be seen from Figure 10, tablet volume and tablet thickness are similar, as might be expected. Compression stress and solid fraction, RTS, applied density, hardness, density at maximum pressure, final density, and maximum density are all located in the same quadrant suggesting that they are closely associated. Interestingly, uncompacted density and recovery are distinct, indicating that they are independent properties showing no similarity to any other properties and will therefore require separate measurements. The overall information from Figure 9 suggests that in future experiments, it may not be necessary to measure all the properties listed above; instead, the number of properties measured can be reduced if they fall into the same quadrant. Figure 10 shows the scores of all the material-associated properties for each compaction force on F1 and F2. Here, the materials under certain compaction forces demonstrating similar properties are separated by the groups formed in the four quadrants. For instance, in the top-right quadrant of Figure 11, the oval region contains M14, M41, M42, M43, M44, M51, M52, M61, M62, M63, M64, M72, M74, and M83. This means that the properties for Benecel CR (M1) under 7.5 kN, Methocel CR (M4) under the compaction force from 15 kN to 7.5 kN, Methocel DC (M5) under 15 kN and 12.5 kN, Metolose 90SH (M6) under 15 kN and 12.5 kN, Metolose 90SH SR (M7) under 12.5 kN and 7.5 kN and BonuCel D (M8) under 10 kN showed a strong similarity in results.

To simplify things further, a plot (Figure 11) for the scores (SF1) on F1 is shown: F1 covers 89.1% variation of the property data. As indicated by the dashed straight line crossing the plot. BonuCel D/Benecel XR, Methocel DC, and Benecel DC have the same score when their corresponding compaction forces are: 7.5 kN, 10 kN, and 12.5 kN, respectively. This means that BonuCel D or Benecel XR under 7.5 kN, Methocel DC under 10 kN and Benecel DC under 12.5 kN will demonstrate similar properties overall. This also suggests that as long as there is a right match between the material properties and the compaction force, some of the HPMC materials studied can be interchangeable.

## 4. Conclusions

The goal of this research was to understand the similarities and differences between equivalent grades of HPMC K100M from different vendors, from a manufacturing perspective, rather than a performance perspective. This study was conducted through the comparison of the flow, particle size, and compaction behaviour of eight polymers from four different companies. The micromeritic analysis showed the CR grades (i.e., not the grades suited to the direct compression process) of the HPMC K100M polymers have smaller particles compared to the DC grades. Due to the larger particle size of the Methocel DC grade compared to other materials evaluated, the flowability analysis indicated its superior flowability characteristics. The hardness and solid fraction analysis showed that the DC grade polymers from the different vendors all demonstrated comparatively lower hardness and solid fraction values relative to the equivalent CR grades. The mechanical properties were also studied under five different compression forces (5 to 15 kN), with the results indicating that the DC grade has relatively lower tabletability, compressibility, and compactability under different compression forces compared to the CR grades. A statistical factor analysis made, however, confirms that under certain conditions of material properties and compression, the grades of HPMC studied from the four different vendors could be potentially used interchangeably. However, care and consideration should be taken, as only single lots of polymer were used from each vendor, and they were assumed to be representative of material from that supplier, and the degree of lot-to-lot variability from each supplier has as yet not been characterised. Additionally, no work on drug incorporation was involved, and the role of polymer performance in dissolution may differ, although it is believed that polymer substitution chemistry and viscosity grade are dominant parameters here, offering control across materials from different sources. 

## Data Availability

The data presented in this study are held by the University of Huddersfield but may be made available, subject to review, on request from a corresponding author.

## Supplementary Materials

The following supporting information can be downloaded at: https://www.mdpi.com/article/10.3390/pharmaceutics15082154/s1: Figure S1. Volume-weighted and arithmetically weighted size, fibre length, and width of Methocel CR (red) and DC (green) grades of HPMC; Figure S2. Volume weighted and arithmetically weighted size, fibre length, and width of Benecel CR (red), DC (green), and XR (blue) grades of HPMC; Figure S3. Volume-weighted and arithmetically weighted size, fibre length, and width of Metolose 90SH (red) and Metolose 90SH SR (green) grades of HPMC; Figure S4. Volume-weighted and arithmetically weighted size, fibre length, and width of Bonucel D HPMC.
