# Supplementary material for: Comparative Evaluation of the Powder and Tableting Properties of Regular and Direct Compression Hypromellose from Different Vendors
Source: Pharmaceutics. 2023 Aug 17;15(8):2154. doi: 10.3390/pharmaceutics15082154 (PMC10459357; doi:10.3390/pharmaceutics15082154)
Supplement: Supplementary file 1 [file pharmaceutics-15-02154-s001.zip › pharmaceutics-2503159-supplementary.pdf]

Comparative Evaluation of the Powder and Tableting Properties of Regular and Direct Compression Hypromellose from Different Vendors

Nihad Mawla [1], Maen Alshafiee [1], John Gamble [2], Mike Tobyn [2], Lande Liu [3], Karl Walton [4], Barbara R. Conway [1], Peter Timmins [1,*] and Kofi Asare-Addo [1,*]

1 Department of Pharmacy, University of Huddersfield, Huddersfield HD1 3DH, UK; nihad.mawla@hud.ac.uk (N.M.); maen.pharm@gmail.com (M.A.); b.r.conway@hud.ac.uk (B.R.C.)
2 Drug Product Development, Bristol Myers Squibb, Moreton, Merseyside CH46 1QW, UK; john.gamble@bms.com (J.G.); mike.tobyn@bms.com (M.T.)
3 Department of Chemical Sciences, University of Huddersfield, Huddersfield HD1 3DH, UK; l.liu@hud.ac.uk
4 EPSRC Future Metrology Hub, University of Huddersfield, Huddersfield HD1 3DH, UK; k.walton@hud.ac.uk
* Correspondence: p.timmins@hud.ac.uk (P.T.); k.asare-addo@hud.ac.uk (K.A.-A.)

Supplementary information

Geometric size

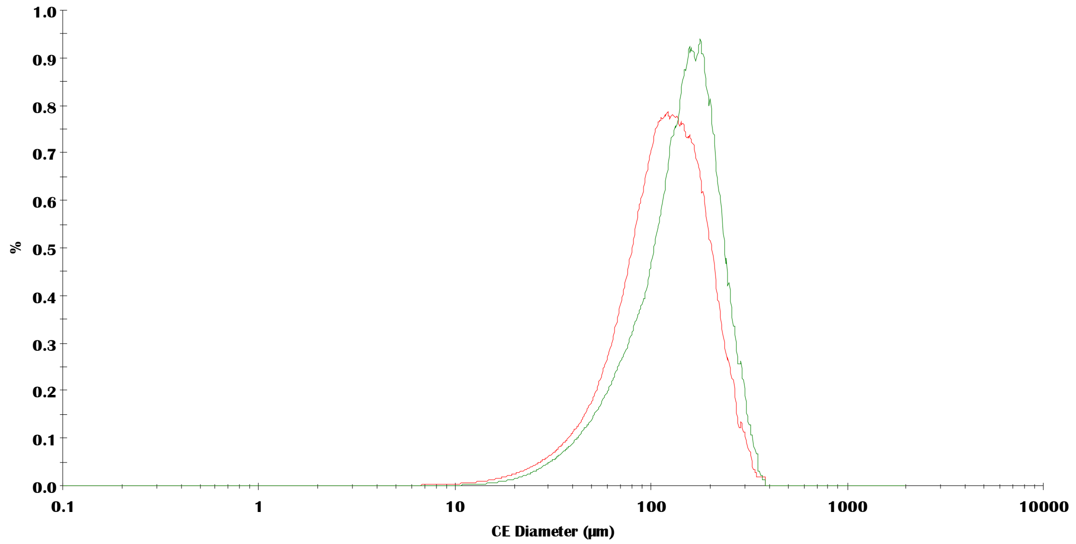

Arithmetic size

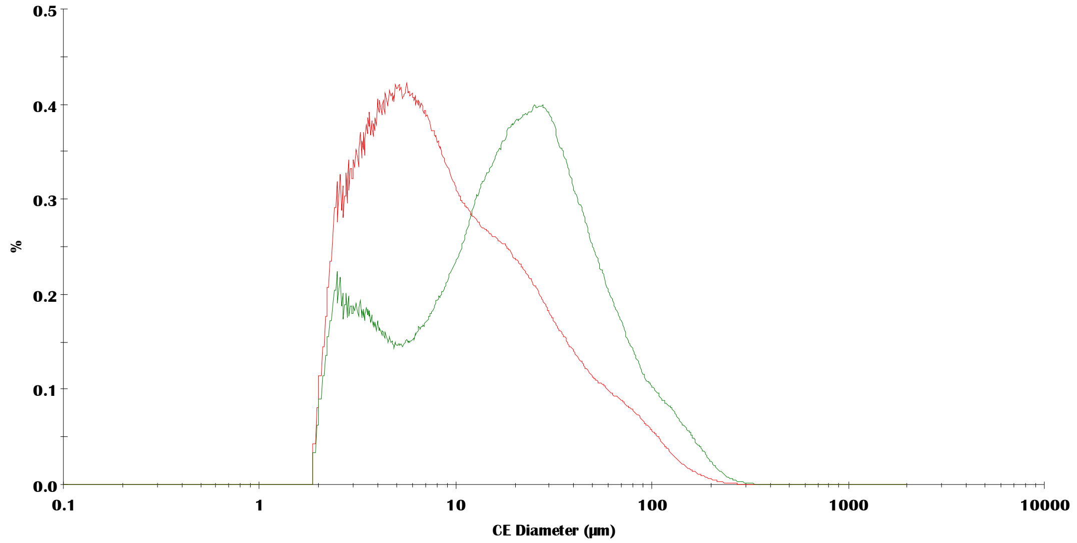

Figure S1. Volume weighted and arithmetically weighted size, fibre length and width of Methocel CR (red) and DC (green) grades of HPMC.

Geometric fibre length

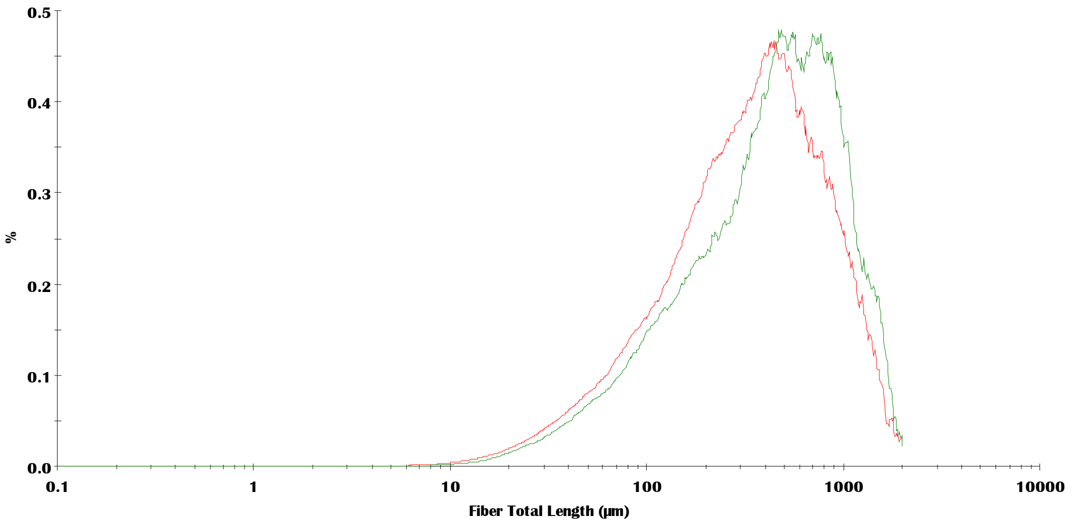

Arithmetic fibre length

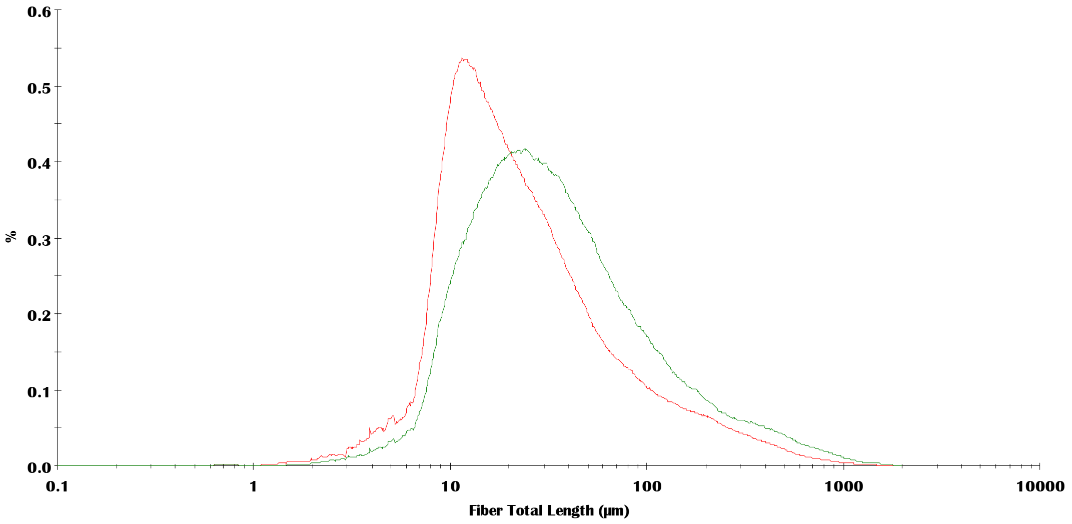

Figure S1 (continued). Volume weighted and arithmetically weighted size, fibre length and width of Methocel CR (red) and DC (green) grades of HPMC.

Geometric fibre width

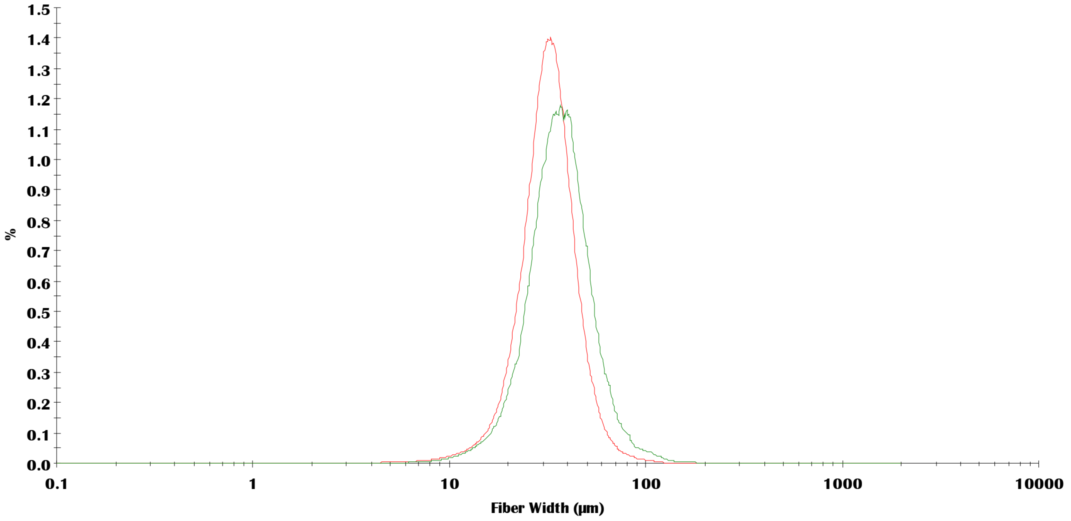

Arithmetic fibre width

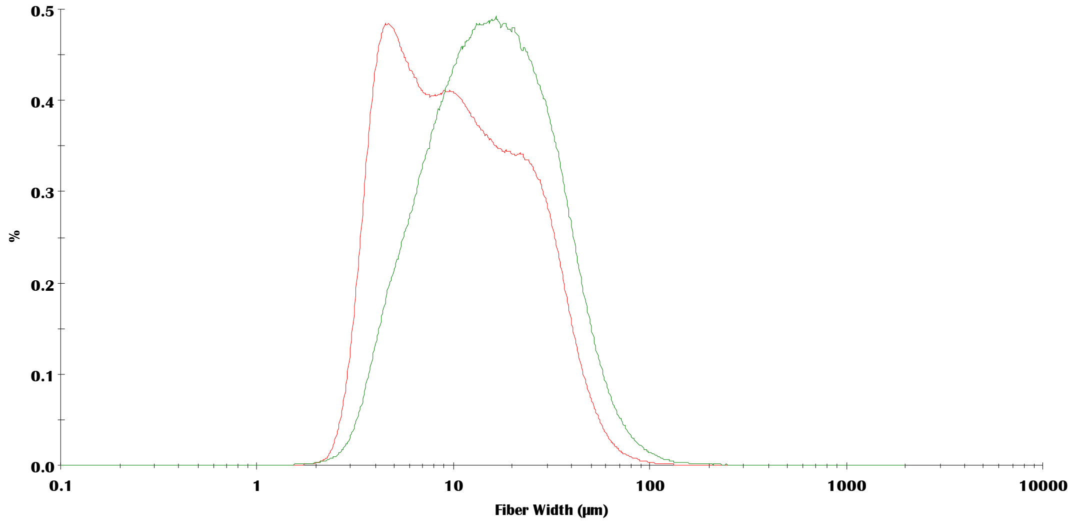

Figure S1 (continued). Volume weighted and arithmetically weighted size, fibre length and width of Methocel CR (red) and DC (green) grades of HPMC.

Geometric size

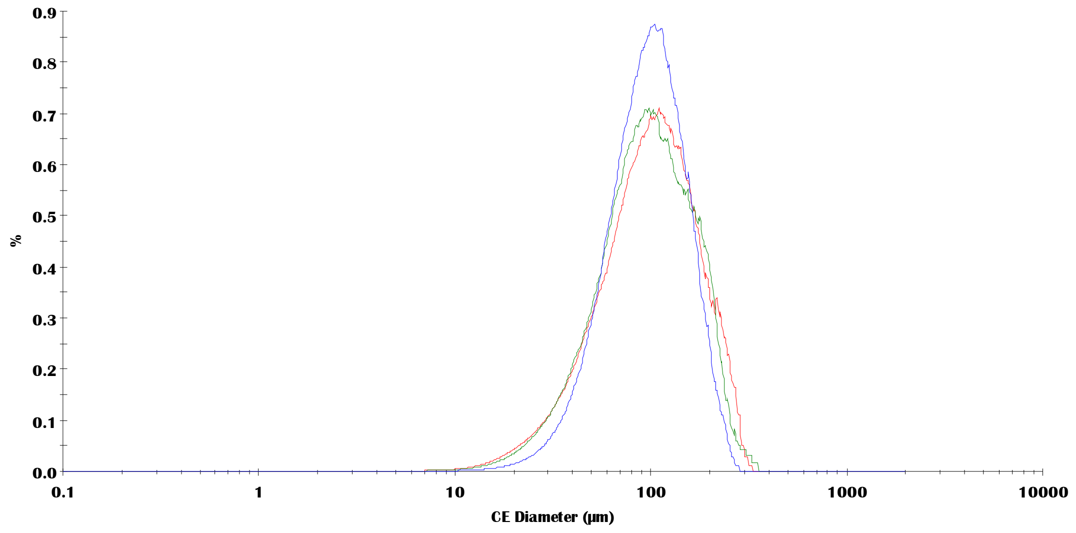

Arithmetic size

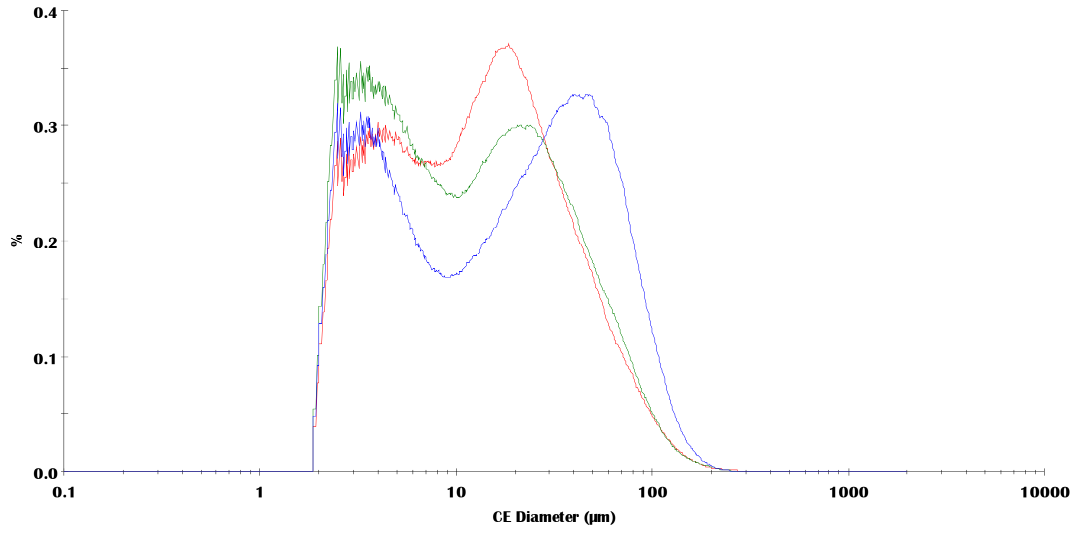

Figure S2. Volume weighted and arithmetically weighted size, fibre length and width of Benecel CR (red), DC (green) and XR (blue) grades of HPMC.

Geometric fibre length

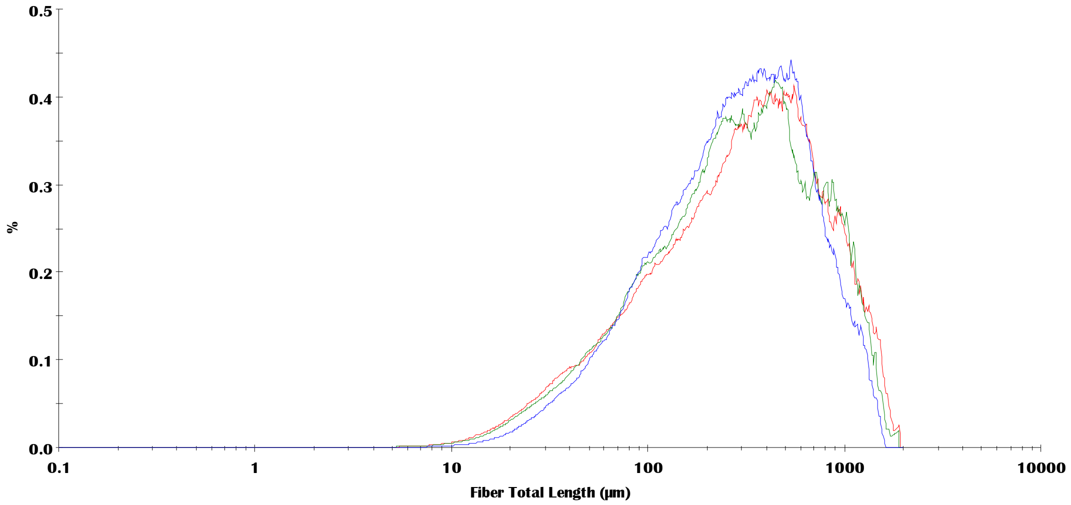

Arithmetic fibre length

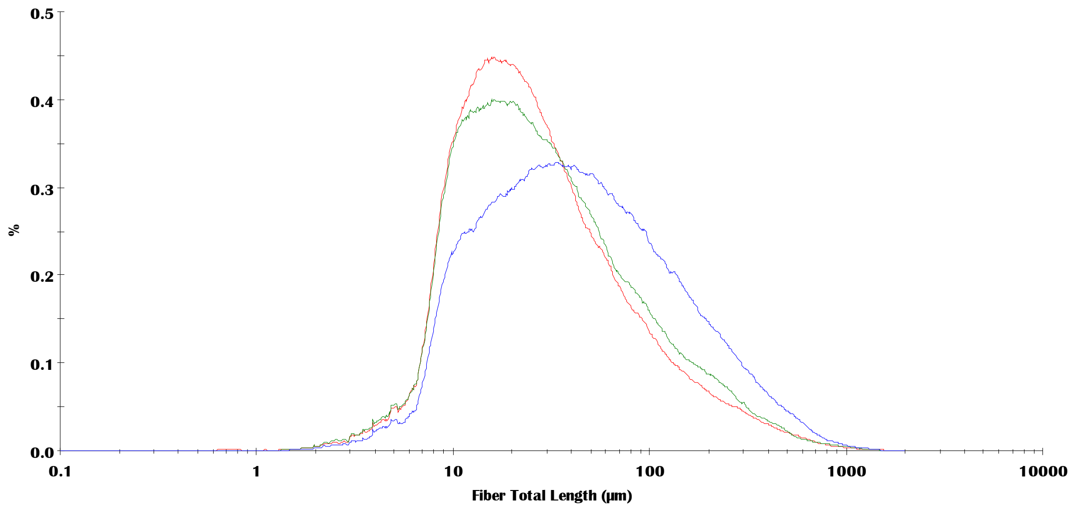

Figure S2 (continued). Volume weighted and arithmetically weighted size, fibre length and width of Benecel CR (red), DC (green) and XR (blue) grades of HPMC.

Geometric fibre width

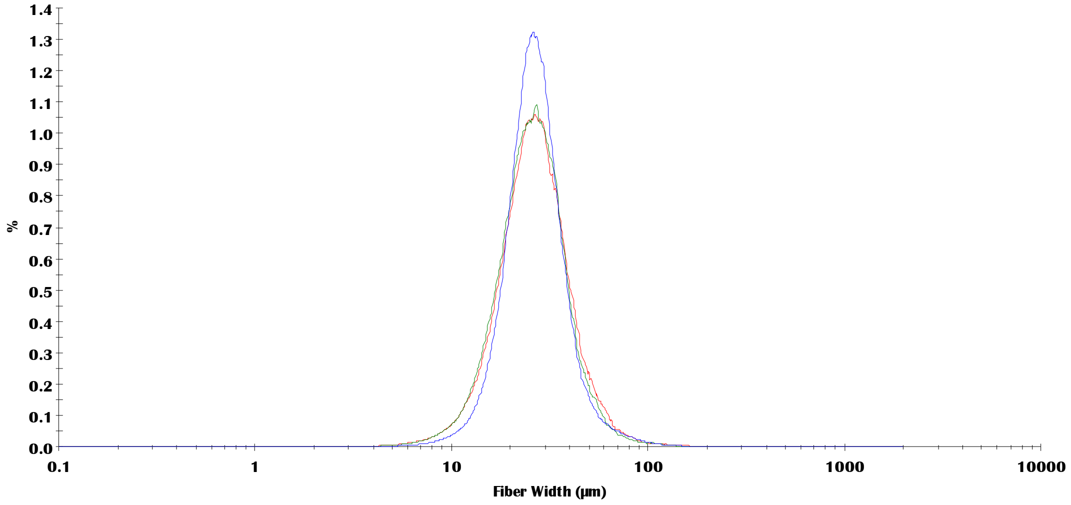

Arithmetic fibre width

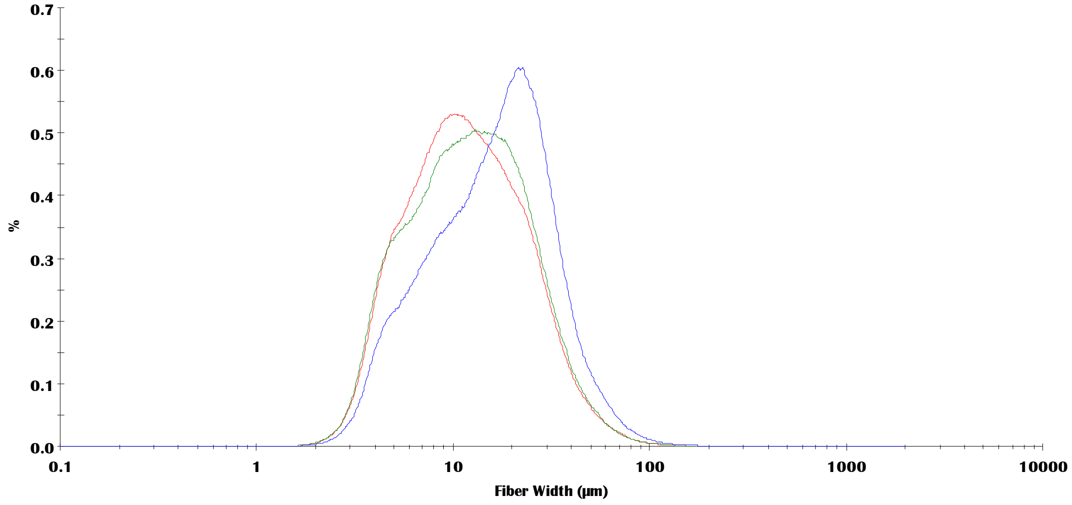

Figure S2 (continued). Volume weighted and arithmetically weighted size, fibre length and width of Benecel CR (red), DC (green) and XR (blue) grades of HPMC.

Geometric size

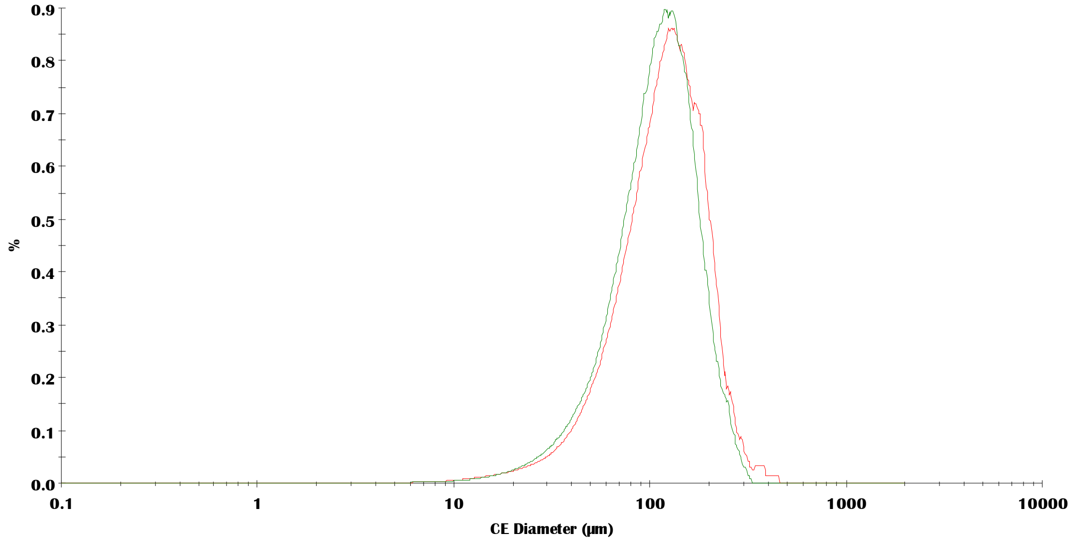

Arithmetic size

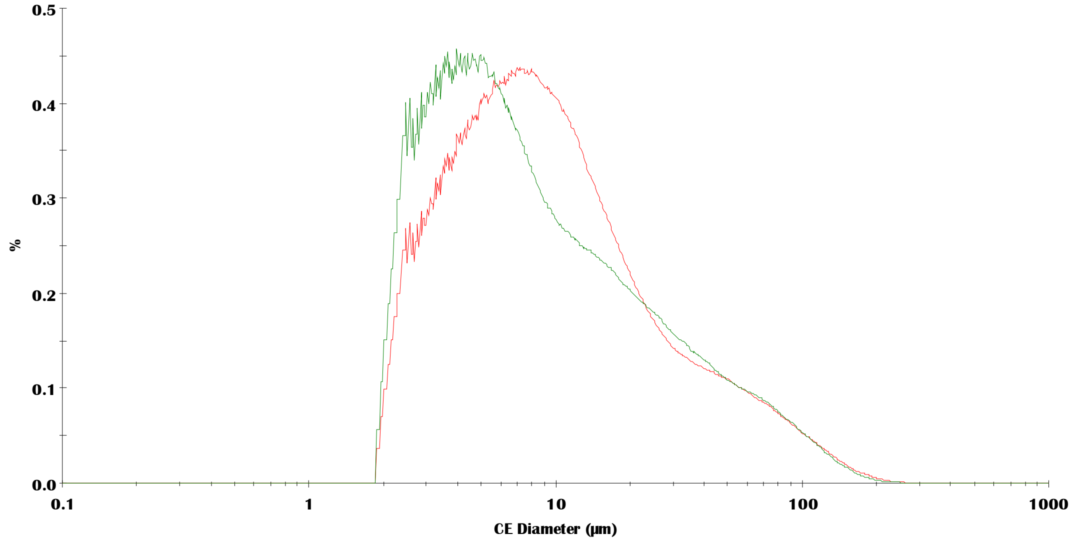

Figure S3. Volume weighted and arithmetically weighted size, fibre length and width of Metolose 90SH (red) and Metolose 90SH SR (green) grades of HPMC.

Geometric fibre length

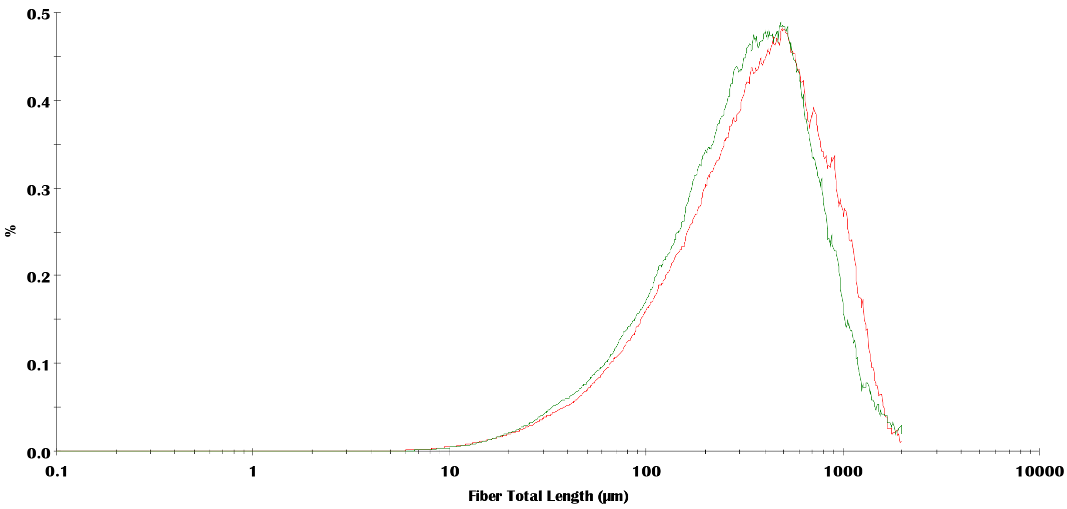

Arithmetic fibre length

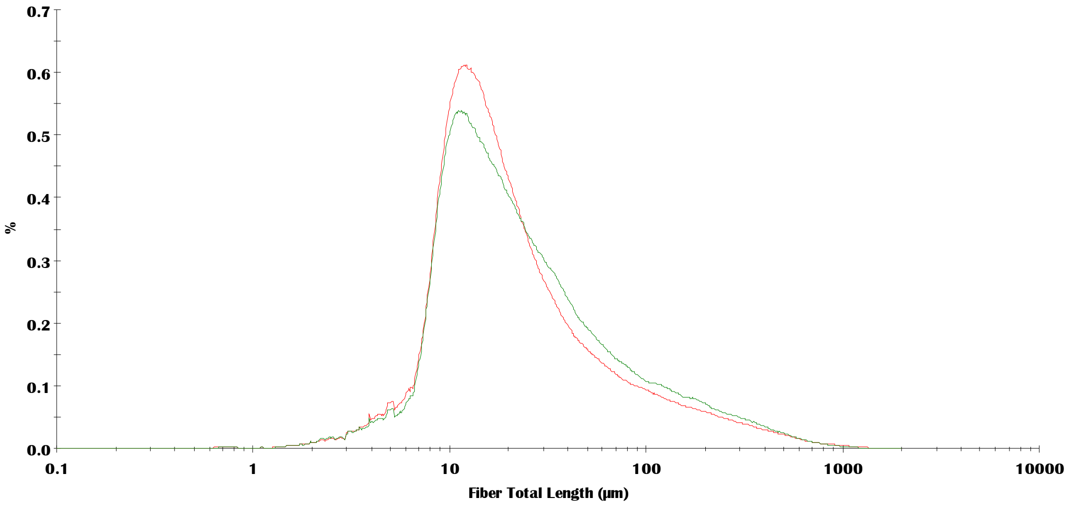

Figure S3 (continued). Volume weighted and arithmetically weighted size, fibre length and width of Metolose 90SH (red) and Metolose 90SH SR (green) grades of HPMC.

Geometric fibre width

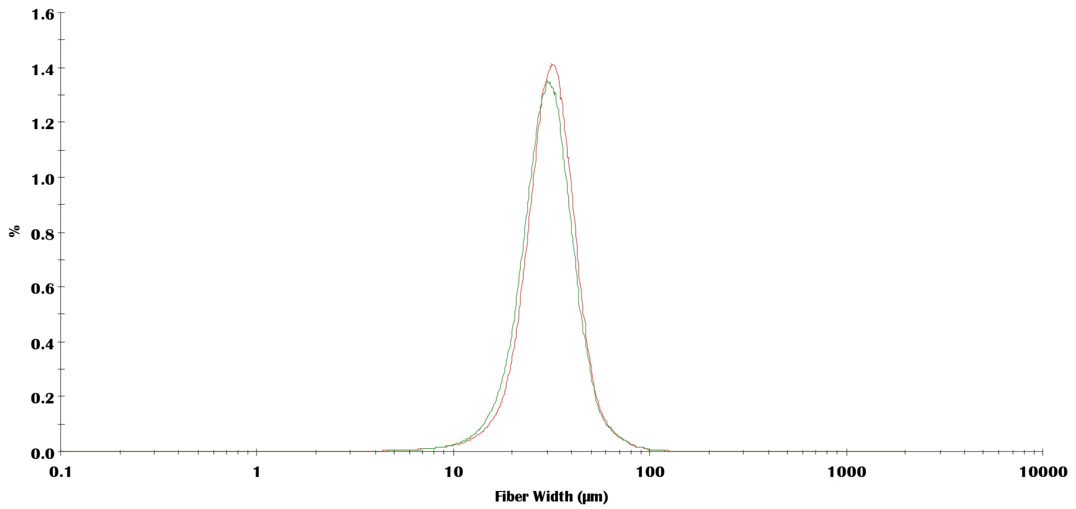

Arithmetic fibre width

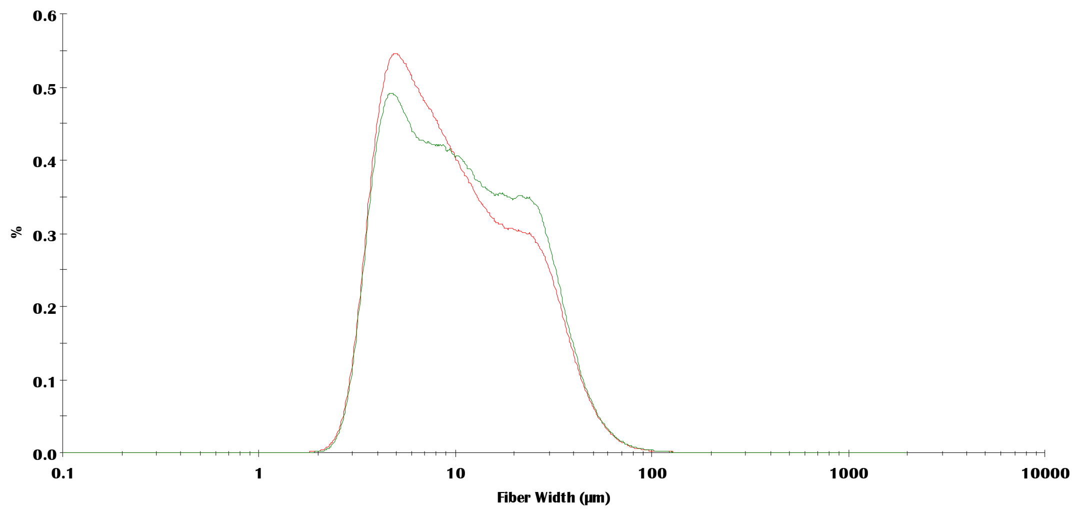

Figure S3 (continued). Volume weighted and arithmetically weighted size, fibre length and width of Metolose 90SH (red) and Metolose 90SH SR (green) grades of HPMC.

Geometric size

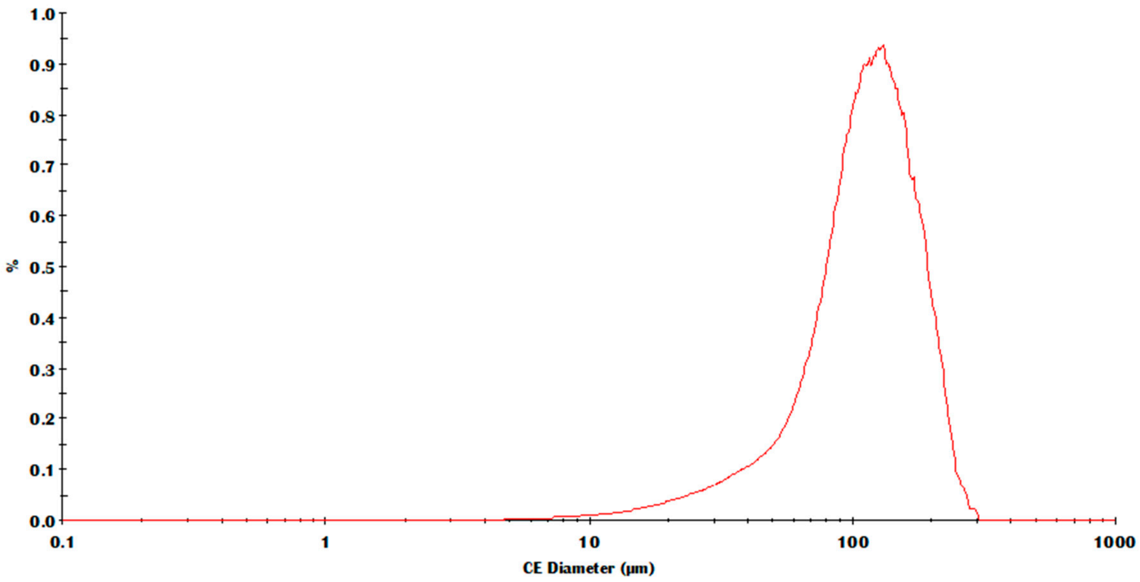

Arithmetic size

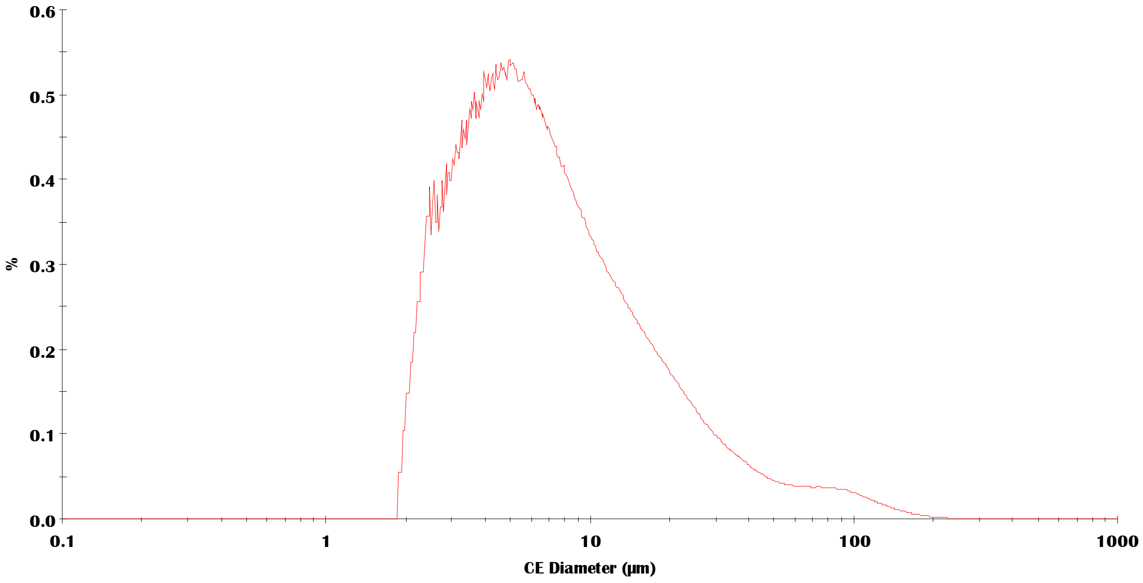

Figure S4. Volume weighted and arithmetically weighted size, fibre length and width of Bonucel D HPMC

Geometric fibre length

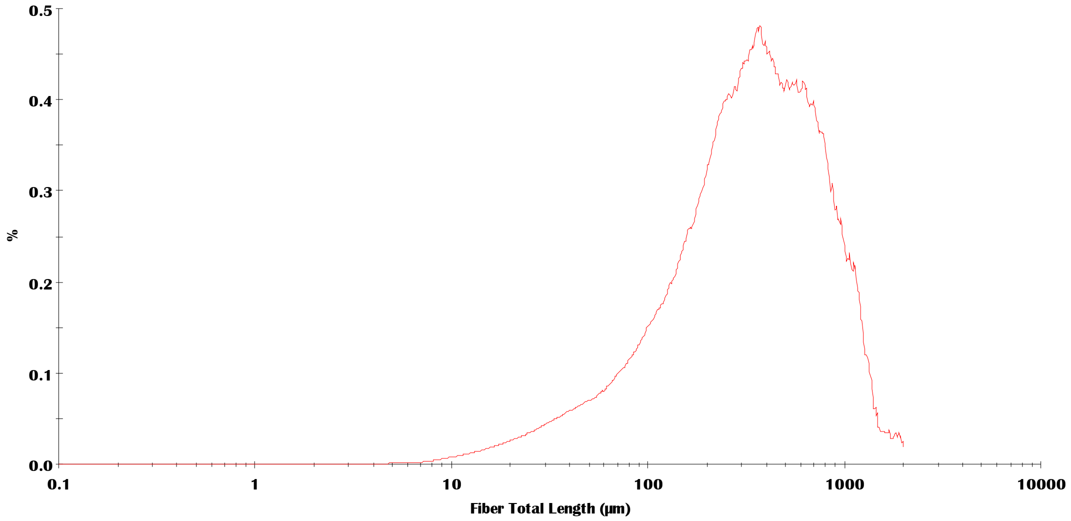

Arithmetic fibre length

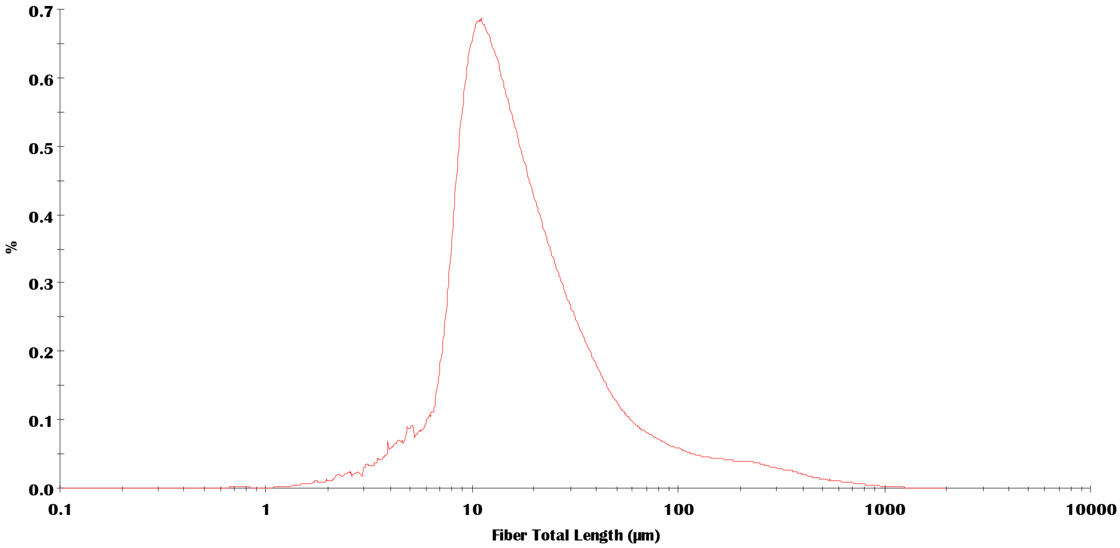

Figure S4 (continued). Volume weighted and arithmetically weighted size, fibre length and width of Bonucel D HPMC

Geometric fibre width

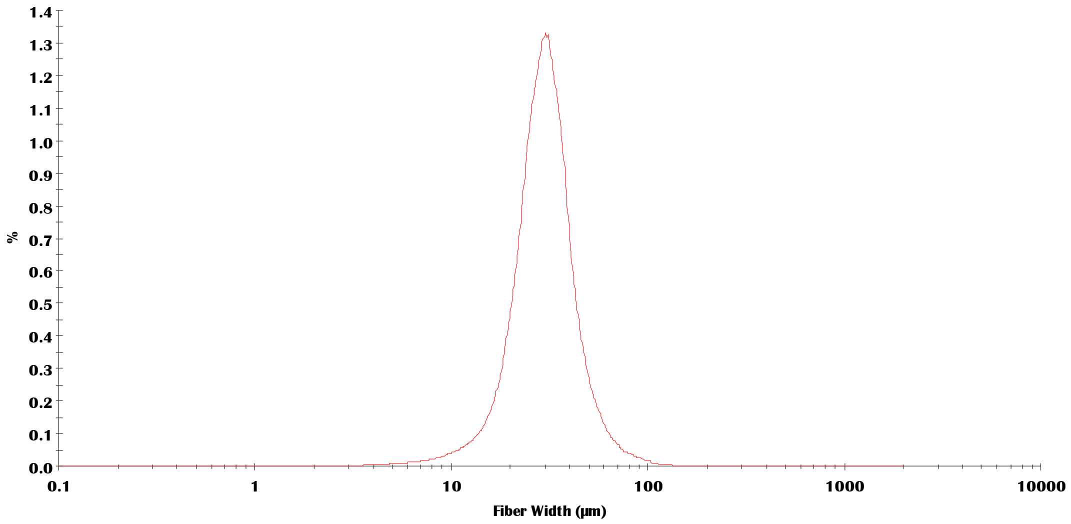

Arithmetic fibre width

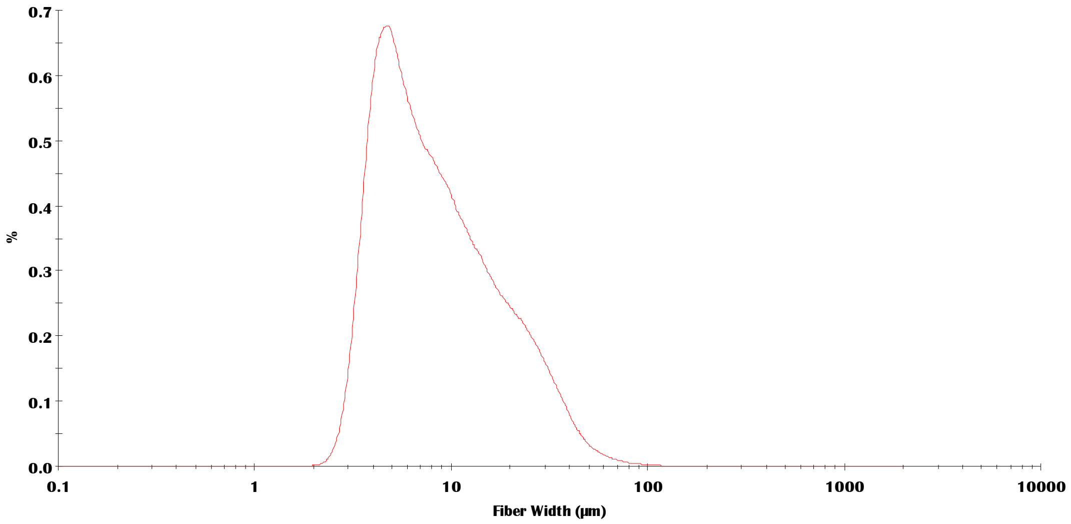

Figure S4 (continued). Volume weighted and arithmetically weighted size, fibre length and width of Bonucel D HPMC
